# Primary Colorectal Tumor Location and Predictors for Metastasis to the Brain

**DOI:** 10.7759/cureus.39735

**Published:** 2023-05-30

**Authors:** William Franceschi, Jonathan Bliggenstorfer, Anuja L Sarode, Meridith Ginesi, Emily Steinhagen, Sharon L Stein

**Affiliations:** 1 Department of General Surgery, Case Western Reserve University School of Medicine, Cleveland, USA; 2 Department of Surgery, University Hospitals Cleveland Medical Center — University Hospitals Research in Surgical Outcomes & Effectiveness Center (UH-RISES), Cleveland, USA

**Keywords:** predictors of metastasis, metastatic brain tumors, adenocarcinoma of colon, metastatic colo-rectal cancer, brain tumors (primary or brain metastasis), colorectal cancer

## Abstract

Introduction

Although rectal cancer is thought to have a higher rate of metastasis to the brain compared with colon cancer, there is limited and contradictory data on the subject. This study aims to determine the prevalence of brain metastasis for colon and rectal cancers (CRC), and to explore associations and predictors of brain metastasis (BM).

Methods

The 2010-2016 National Cancer Database (NCDB) was queried for patients with stage IV CRC. Patients with missing data on site of metastasis and primary tumor location were excluded. Chi-square test was used for categorical data and multivariate logistic regression analysis was performed to evaluate the predictors of BM.

Results

Of 108,540 stage IV CRC patients, the prevalence of BM was 1.21% from the right colon, 1.29% from the left colon, and 1.59% from the rectal adenocarcinoma (p<0.001). The presence of lung, bone, and liver metastases were the strongest predictors for BM. Bone and lung metastases increased the odds for BM by 3.87 (95% CI: 3.36-4.46) and 3.38 (95% CI: 3.01-3.80), respectively while the presence of liver metastasis decreased odds for BM by 55% (OR: 0.45; 95% CI: 0.40-0.50). On multivariate analysis, primary tumor location was not predictive of BM.

Discussion

This study helps to characterize the prevalence and associations of BM from CRC using the NCDB. The correlation between BM and bone and lung metastases, along with negative association of liver metastasis further supports the hypothesis of systemic transmission of tumor cells. Further identification of predictors and correlations with BM may help guide surveillance among patients with advanced CRC.

## Introduction

Although relatively rare, colorectal cancer (CRC) that metastasizes to the brain is associated with very poor prognosis [1-4]. Early detection of brain metastases (BM) can guide treatment and lead to improved survival outcomes. However, current practice is to wait for the onset of neurologic symptoms before screening, as the efficacy and financial viability of early screening is unknown [5,6]. Identifying stage IV CRC patients at greatest risk for BM may therefore assist in the development of a risk stratification tool to target screening, leading to more timely diagnosis and intervention.

Risk factors that lead to the development of BM are not well described, but are commonly believed to occur in association with long survival, long-standing pulmonary metastases, and left-sided tumors [6-10]. There is also clinical suspicion that rectal cancers metastasize to the brain more frequently than colon cancers, possibly due to differences in peritoneal coverage and venous drainage of the rectum and colon [11,12]. However, data on patterns and risk factors for metastases to the brain are limited and insufficient [6]. An autopsy study of 1675 patients found a higher incidence of BM in rectal cancer compared to colon cancer (5.0 vs 2.6%) [13]. Two retrospective review studies found a positive association between BM and rectal cancer, but neither study’s findings reached statistical significance [14,15]. Given these inconsistent findings regarding BM patterns between colon and rectal cancers, more investigation is required to elucidate the prevalence and risk factors for BM among these patients.

This epidemiologic study seeks to define the relative prevalence of BM among stage IV colon and rectal cancer using a large national database, as well as to determine predictors for BM in each patient population. We hypothesized that there will be a higher prevalence of BM among patients with rectal cancer compared to colon cancer.

## Materials and methods

**Figure 1:**
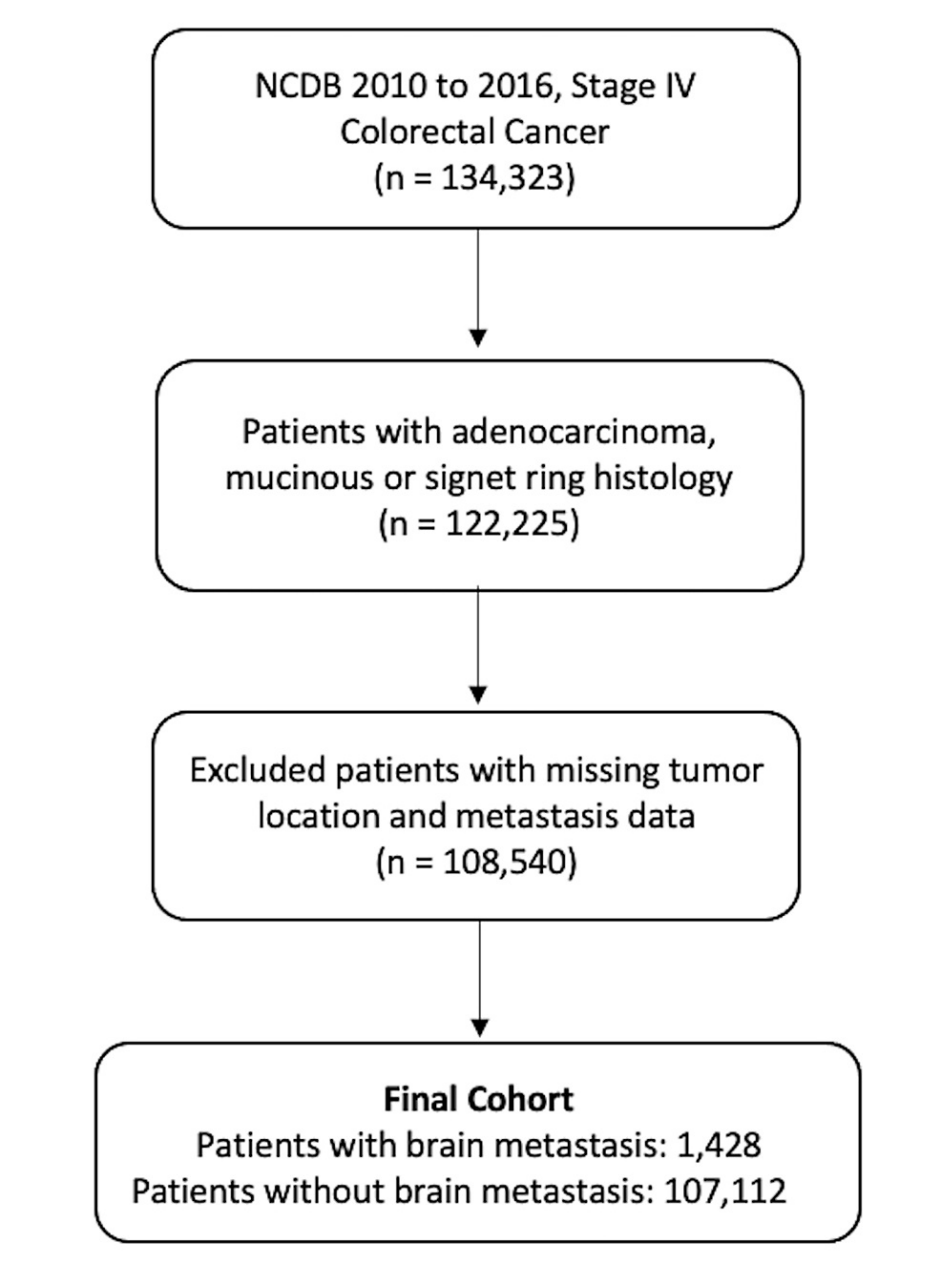
Inclusion and exclusion criteria

The National Cancer Database (NCDB) is a clinical oncology database sponsored by the American College of Surgeons and the American Cancer Society. The NCDB tracks clinical and outcomes data for neoplastic diseases, representing approximately 70% of all newly diagnosed cancers in the United States. The 2010-2016 NCDB for colon and rectal cancer was queried for patients with stage IV CRC [16]. Patients with missing data on metastasis sites and primary tumor location were excluded (Figure 1). This study was reviewed by the University Hospitals Cleveland Medical Center Institutional Review Board (IRB) and was determined to be non-human subject research.

Definitions

Following inclusion and exclusion criteria, patients were divided into two cohorts, presence of BM and absence of BM. Primary tumor location, demographics, staging, and additional sites of metastasis were assessed for each cohort.

Statistical analysis

The group comparison analyses were performed using Student T-tests for continuous variables and Chi-square tests for categorical variables. Statistically significant variables including primary tumor location, race, insurance status, facility type, clinical N, grade, and lung, bone, and liver metastases, were included in a multivariable logistic regression model to identify predictors for BM. Kaplan-Meier curves were generated for BM patients based upon primary tumor location. Log-rank tests were utilized to evaluate the survival distribution between these groups.

All the complete case analyses were performed in SAS version 9.4 statistical software (SAS Institute Inc., Cary, NC), with two-tailed p<0.05 considered statistically significant.

## Results

Prevalence of brain metastasis

The prevalence of BM for all stage IV CRC patients was 13.2 per 1,000 patients (n=1,428). Overall, right and left colon cancer patients were less likely to have BM than rectal cancer patients. The prevalence of BM for right colon, left colon, and rectal adenocarcinoma was 12.1 (n=566), 12.9 (n=520), and 15.9 in 1,000 patients (n=342) (p<0.001), respectively (Table 1).

**Table 1:** Demographic and clinical characteristics of stage IV colon and rectal cancer patients (n=108,540)

Variables				Presence of Brain Metastasis				
				No n=107112 (98.68%)	Yes n=1428 (1.32%)	P value		
Tumor Location	Right Colon			46608 (98.79)	566 (1.21)	<0.001		
	Left Colon			39865 (98.71)	520 (1.29)			
	Rectal			21205 (98.41)	342 (1.59)			
Age (mean)				63.56 (±13.80)	63.20 (±12.69)	0.294		
Sex	Male			57514 (53.70)	758 (53.08)	0.644		
	Female			49598 (46.30)	670 (46.92)			
Race	White			85771 (80.08)	1200 (84.03)	0.001		
	Black			15661 (14.62)	166 (11.62)			
	Other/Unknown			5680 (5.30)	62 (4.34)			
Spanish/Hispanic	Non-Spanish/Hispanic			97298 (90.84)	1296 (90.76)	0.814		
	Spanish/Hispanic Origin			6647 (6.21)	86 (6.02)			
	Unknown			3167 (2.96)	46 (3.22)			
Charlson Deyo	Zero			79745 (74.45)	1075 (75.28)	0.475		
	One or more			27367 (25.55)	353 (24.72)			
Area	Metro			85197 (81.52)	1120 (80.23)	0.317		
	Urban			16811 (16.08)	245 (17.55)			
	Rural			2507 (2.40)	31 (2.22)			
Insurance Status	Not Insured			5793 (5.41)	99 (6.93)	0.003		
	Private Insurance / Managed Care			40683 (37.98)	504 (35.29)			
	Medicaid			10103 (9.43)	157 (10.99)			
	Medicare			47712 (44.54)	626 (43.84)			
	Other Government			1205 (1.12)	25 (1.75)			
	Insurance Status Unknown			1616 (1.51)	17 (1.19)			
Facility Type	Community Cancer Program			11513 (11.24)	118 (8.57)	0.007		
	Comprehensive Community Cancer Program			41989 (40.98)	554 (40.23)			
	Academic/Research Program			35115 (34.27)	506 (36.75)			
	Integrated Network Cancer Program			13834 (16.50)	199 (14.45)			
Clinical N	0			41641 (41.15)	507 (36.90)	0.005		
	1			23458 (23.18)	319 (23.22)			
	2			10037 (9.92)	152 (11.06)			
	Unknown			26058 (25.75)	396 (28.82)			
Clinical T	1			6925 (6.98)	113 (8.37)	<0.001		
	2			2150 (2.17)	31 (2.30)			
	3			17219 (17.35)	210 (15.56)			
	4			14517 (14.63)	121 (8.96)			
	Unknown			58446 (58.88)	875 (64.81)			
Histological Grade	Grade I: Well differentiated			6261 (5.85)	62 (4.34)	<0.001		
	Grade II: Moderately differentiated			53616 (50.06)	562 (39.36)			
	Grade III: Poorly differentiated			20536 (19.17)	297 (20.80)			
	Grade IV: Undifferentiated; anaplastic			3112 (2.91)	39 (2.73)			
	Unknown			23587 (22.02)	468 (32.77)			
Bone Metastasis	Yes			5075 (4.74)	301 (21.08)	<0.001		
	No			101823 (95.06)	1103 (77.24)			
	Unknown			214 (0.20)	24 (1.68)			
Lung Metastasis	Yes			23605 (22.04)	739 (51.75)	<0.001		
	No			82577 (77.09)	668 (48.78)			
	Unknown			930 (0.87)	21 (1.47)			
Liver Metastasis	Yes			75835 (70.80)	786 (55.04)	<0.001		
	No			30970 (28.91)	628 (43.98)			
	Unknown			307 (0.29)	14 (0.98)			

Analysis of demographic and clinical characteristics

Patients with and without BM significantly differed on the basis of clinicodemographic characteristics (Table 1). Patients with BM were more likely to have bone metastases (21.1% BM vs 4.7% without BM, p<0.001), lung metastases (51.8% BM vs 22.0% without BM, p<0.001), clinical N2 (11.2% BM vs 9.9% without BM, p=0.005), and poorly differentiated tumors (20.8% BM vs 19.2% without BM, p<0.001) than patients with stage IV disease and no BM. Patients with BM were also more likely to be uninsured (6.9% BM vs 5.4% without BM), on Medicaid (11.0% BM vs 9.4% without BM, p=0.003), and treated at an academic/research center (36.8% BM vs 34.3% without BM, p=0.007). Conversely, patients with BM were less likely to present with liver metastases (55.0% BM vs 70.8% without BM, p<0.001). Patients with BM were also less likely to be black (11.6% BM vs 14.6% without BM, p=0.001) or have private insurance (35.3% BM vs 38.0% without BM, p=0.003). Although nodal disease was associated with BM (36.9% clinical N0 BM vs 41.1% clinical N0 without BM, p=0.005), more than one-third of BM patients had no nodal involvement.

Predictors of brain metastasis - Multivariable analysis

Notably, primary tumor location was not identified as being a significant predictor for BM on multivariate analysis. Right-sided colon cancer or left-sided colon cancer did not significantly increase or decrease the odds of BM when compared to rectal adenocarcinoma (Table 2). Presence of bone metastases (OR: 3.87; 95% CI: 3.36-4.46), presence of lung metastases (OR: 3.38; 95% CI: 3.01-3.80), poorly differentiated grade III tumors (OR: 1.43; 95% CI: 1.07-1.91), and clinical N2 disease (OR: 1.37; 95% CI: 1.12-1.67) positively predicted BM. Liver metastases (OR: 0.45; 95% CI: 0.40-0.50) and African American ethnicity (OR: 0.74; 95% CI: 0.63-0.88) negatively predicted BM.

**Table 2:** Multivariable logistic regression for predictors of brain metastasis (n = 97,859)

Variables		Odds Ratio	95% Confidence Interval		
Tumor Location	Left Colon vs Rectal	1.12	0.96 - 1.30
	Right Colon vs Rectal	1.08	0.93 - 1.26
Sex	Female vs Male	1.06	0.95 - 1.18
Race	Black vs White	0.74	0.63 - 0.88
	All other vs White	0.79	0.61 - 1.03
Area	Rural vs Metro	0.96	0.67 - 1.39
	Urban vs Metro	1.13	0.97 - 1.30
Insurance	None vs Insured	1.19	0.95 - 1.49
	Unknown vs Insured	0.80	0.48 - 1.34
Facility Type	All other vs Academic/Research	0.91	0.81 - 1.02
Clinical N Stage	1 vs 0	1.10	0.95 - 1.28
	2 vs 0	1.37	1.12 - 1.67
	Unknown vs 0	1.15	1.00 - 1.32
Grade	2 vs 1	1.06	0.80 - 1.40
	3 vs 1	1.43	1.07 - 1.91
	4 vs 1	1.25	0.81 - 1.92
	Unknown vs 1	1.70	1.29 - 2.26
Bone Metastasis	Yes vs No	3.87	3.36 - 4.46
	Unknown vs No	6.79	4.19 - 11.01
Lung Metastasis	Yes vs No	3.38	3.01 - 3.80
	Unknown vs No	2.05	1.25 - 3.36
Liver Metastasis	Yes vs No	0.45	0.40 - 0.50
	Unknown vs No	1.38	0.75 - 2.55

Survival analysis

**Figure 2:**
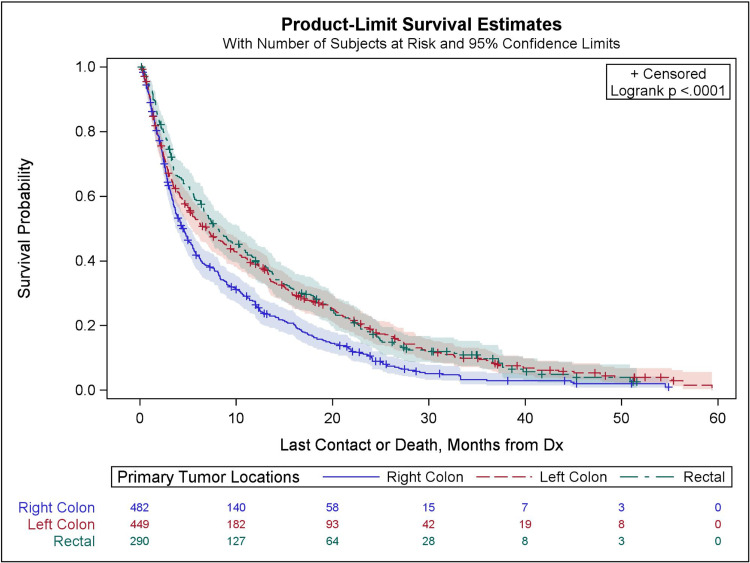
Kaplan-Meier survival curve estimates for brain metastasis patients by primary tumor location

Kaplan-Meier survival curves for patients with BM based on primary tumor location are shown in Figure 2. BM patients from right-sided colon cancer had the poorest survival rates. BM patients from left-sided colon cancer and rectal adenocarcinoma showed no statistically significant difference in survival rates. The 12-month survival rates for BM patients with right-sided colon cancer, left-sided colon cancer, and rectal cancer were 26.4%, 39.0%, and 40.0%, respectively. The 24-month survival rates for BM patients with right-sided colon cancer, left-sided colon cancer, and rectal were 11.3%, 19.4%, and 18.1%, respectively (p<0.001).

## Discussion

This study evaluated the epidemiology of patients with BM and colon and rectal cancer using the NCDB. Prevalence of BM was highest among rectal adenocarcinoma patients compared to right or left-sided colon cancer, supporting long-standing clinical suspicion. Multivariable analysis, however, demonstrated no association of the location of the primary tumor with the occurrence of BM. Additionally, strong correlations were noted with bone and lung metastases, but the presence of liver metastases was negatively correlated with the incidence of BM. Black race was associated with a lower frequency of BM.

Our analysis adds to the literature by evaluating the incidence and predictors for BM among a large representative patient population. While incidence and predictors for BM among stage IV CRC patients have not been well studied, a previous analysis of the SEER database found the relative prevalence of BM to be similar to the current study: 12.8 in 1,000 patients (n=186), 11.5 in 1,000 patients (n=156), and 14.4 in 1,000 patients (n=86) in right colon cancer, left colon cancer, and rectal adenocarcinoma respectively [17]. Their data did not, however, reach statistical difference (p=0.2). This may be explained by the smaller patient population (38,783 vs 108,540 metastatic CRC patients) of the SEER data. However, given the size of the databases and the fact that multivariable analysis in the current study failed to show the location as a predictive variable, the location may not be as important at predicting brain metastases as the co-location of other metastases.

This data supports a correlation of BM with bone and lung metastases on multivariable analysis. These sites are all presumed to be associated with hematogenous spread via the systemic circulation. On the other hand, liver metastases, more indicative of hematogenous spread via the portal circulation, were significantly associated with a decreased odds of BM. These indicators of systemic versus portal spread were the strongest predictors for BM, suggesting that hematogenous spread via systemic circulation is the predominant mechanism for metastasis to the brain. These data augment the findings of prior studies, which have found that BM from CRC spread hematogenously and present with multiple lesions (commonly lung and bone) and neurological symptoms [18,19]. Interestingly, more than one-third of BM patients had no nodal involvement, further indicating that metastases to the brain are predominately hematogenous.

Some of the factors associated with BM, such as Clinical N2 staging and poorly-differentiated grade III histology, were expected. In general, high-grade tumors with increased nodal involvement are more likely to metastasize. African American ethnicity, a negative predictor of BM, requires further research. Prior studies have described an increased incidence of right colon cancer in African American populations and hypothesized that African American patients with CRC have a higher frequency of microsatellite stability [20,21]. The differing genetic profiles, histology, and patterns of metastasis of these malignancies may explain the negative association between African American ethnicity and BM but a complete understanding in the context of BM remains unknown.

Limitations

There are some intrinsic limitations to the NCDB, including the accuracy of data entry and missing clinicodemographic data, that affected this study’s analysis. Clinical T stage was missing for the majority of patients, likely rendering univariate and multivariate analysis of clinical T and BM invalid. The NCDB also does not include temporal information regarding metachronous or synchronous presentations of stage IV disease. As a result, this study was unable to determine the order of metastasis and whether this may predict the occurrence of brain metastases. This limited the analysis of metastatic patterns, especially when comparing hematogenous spread via the portal or systemic circulation. In addition, information such as clinical staging, genetic profiles, and date of death was missing for a significant portion of these patients, limiting the applicability of these metrics in the analysis.

Future directions

Further research and cost-benefit analysis are needed to guide additional screening for stage IV CRC patients at the time of diagnosis. While CRC patients standardly receive screening CT imaging of the chest, abdomen, and pelvis at the time of diagnosis, imaging of the head is currently not recommended due to the low incidence of BM, despite improvement in survival outcomes and treatment options with early detection of BM [5,22]. Our study has identified lung and bone lesions as significant predictors for BM that may be applicable in early screening for BM in stage IV CRC patients. Further analysis should include cost evaluation of screening for brain metastases in patients with patterns concerning for progression to brain metastases.

## Conclusions

This study utilized the most comprehensive database available to determine the prevalence and risk factors for BM from CRC. This study found that rectal adenocarcinoma is not a significant predictor for BM when compared to right- or left-sided colon cancers. However, lung and bone metastases significantly increased the odds of the presence of BM, indicating that hematogenous spread via systemic circulation plays a predominant role in the occurrence of BM among patients with stage IV CRC.
